# The NTD Supply Chain Forum—Strengthening the backbone of NTD programs

**DOI:** 10.1371/journal.pntd.0008818

**Published:** 2020-11-05

**Authors:** Ashley A. Souza, Cassandra Holloway, Tijana Williams

**Affiliations:** 1 Neglected Tropical Diseases Support Center, Task Force for Global Health, Atlanta, Georgia, United States of America; 2 Children Without Worms, Task Force for Global Health, Atlanta, Georgia, United States of America; 3 GlaxoSmithKline, Global Health Unit, Neglected Tropical Diseases, Brentford, United Kingdom; London School of Hygiene and Tropical Medicine, UNITED KINGDOM

## Abstract

Global programs targeting 5 preventive chemotherapy neglected tropical diseases (PC-NTDs) have scaled up rapidly in recent decades due, in large part, to the generous drug donations from 6 pharmaceutical companies—Eisai; Johnson & Johnson (J&J); GlaxoSmithKline (GSK); Merck & Co., Inc., Kenilworth, New Jersey, United States of America (MSD); Merck KgaA; and Pfizer. Today, the scale of the PC-NTD drug donation programs is staggering. Nearly 15 billion tablets have been manufactured, packaged, shipped, and distributed in order to reach the people in need. The supply chains established to support such massive operations are enormously complex. Here, we describe a unique public–private partnership that was formed to bring together supply chain expertise to overcome the critical challenges associated with such large-scale production and delivery of donated pharmaceutical products.

## Author summary

Neglected tropical diseases (NTDs) are a diverse group of diseases that disproportionately affect the world’s disadvantaged populations. Five of the diseases—lymphatic filariasis (LF), schistosomiasis (SCH), soil-transmitted helminthiasis (STH), onchocerciasis (ONC), and trachoma (TRA)—are collectively known as the preventive chemotherapy NTDs (PC-NTDs) because the intervention strategy for each is based on delivery of preventive treatment to the entire eligible, at-risk population via mass drug administration (MDA). The global programs targeting these diseases are made possible through the generous donation of drugs from 6 pharmaceutical companies: Eisai; Johnson & Johnson (J&J); GlaxoSmithKline (GSK); Merck & Co., Inc., Kenilworth, New Jersey, United States of America (MSD); Merck KgaA; and Pfizer. Today, the scale of the PC-NTD drug donation programs is staggering. Nearly 15 billion tablets have been manufactured, packaged, shipped, and distributed. The supply chains established to support such massive operations are enormously complex. The Coronavirus Disease 2019 (COVID-19) pandemic has thrust the importance of strong supply chains into global focus like never before. It is therefore an opportune moment to recognize the supply chains that form the backbone of global PC-NTD programs and the unique public–private partnership that was formed to strengthen them.

## NTDs and the global programs targeting them

Neglected tropical diseases (NTDs) affect more than 2.7 billion people in 149 countries [1]. They cause significant long-term morbidity, including visual, physical, and cognitive impairment that prevents children from attending school and adults from earning wages, thus costing developing countries billions of dollars annually. In 2012, the London Declaration on NTDs was signed and endorsed to solidify commitments for the control, elimination, or eradication of 10 NTDs by 2020 [2]. Five of the diseases—lymphatic filariasis (LF), schistosomiasis (SCH), soil-transmitted helminthiasis (STH), onchocerciasis (ONC), and trachoma (TRA)—are collectively known as the preventive chemotherapy NTDs (PC-NTDs) because the intervention strategy for each is based on delivery of preventive treatment to the entire eligible, at-risk population via mass drug administration (MDA).

The drugs required to treat the PC-NTDs are of relatively low cost; however, the cumulative expense of purchasing and transporting drugs and administering MDA to the entire at-risk population generally kept it beyond the reach of most endemic countries.

In 1987, Merck & Co., Inc., Kenilworth, New Jersey, United States of America (MSD) announced that it would donate Mectizan (Ivermectin) through the Mectizan Donation Program for the treatment of ONC for as long as necessary [3]. This commitment ushered in an era of new hope for the fight against NTDs. By 2010, drug donations were in place for each of the 5 PC-NTDs. Table 1 summarizes the commitments and the number of treatments donated to date.

**Table 1:** Summary of NTD drug donation commitments.

Company	Disease	Drug	Commitment(s)	Number of treatments donated (as of January 1, 2020)
Merck & Co., Inc., Kenilworth, NJ, USA (MSD)	ONC, LF	Mectizan (ivermectin)	• 1987 –Donation of Mectizan for ONC for as long as needed [3] • 1998 –Donation of Mectizan for LF in LF/ONC co-endemic areas for as long as needed [6] • 2017–100 million treatments per year for LF, where ONC is not endemic, through 2025 [6]	3.4 billion [7]
GlaxoSmithKline	LF, STH	Albendazole	• 1997–600 million albendazole tablets per year for LF until the disease is eliminated as a public health problem [8] • 2010 –Up to 400 million additional albendazole treatments per year for treatment of STH in school-age children annually through 2020 [9]	9 billion
Pfizer	TRA	Azithromycin	• 1998–10 million doses per year [10] • 2016–100 million doses per year • 2018 –Commitment extended to provide the quantity approved by the Trachoma Expert Committee through 2025 [11]	897 million [11]
Johnson & Johnson	STH	VERMOX, VERMOX CHEWABLE (mebendazole)	• 2006–50 million doses annually [12] • 2012–200 million doses annually [13] • 2019–200 million VERMOX Chewable doses annually for the period 2021–2025 for an additional billion doses [13]	1.5 billion
Merck KgaA	SCH	Praziquantel	• 2007–200 million tablets donated over 10 years [14] • 2017–250 Million tablets annually [15] • 2019 –MOU signed to continue commitment of 250 million tablets annually [15]	400 million [15]
Eisai	LF	Diethylcarbamazine (DEC)	• 2010–2.2 billion tablets for treatment of LF through 2020 [16] • 2017 –Commitment extended until LF elimination is complete [16]	760 Million [16]

DEC, Diethylcarbamazine; LF, lymphatic filariasis; MOU, memorandum of understanding; NTD, neglected tropical disease; ONC, onchocerciasis; SCH, schistosomiasis; STH, soil-transmitted helminthiasis; TRA, trachoma.

Today, the scale of the PC-NTD drug donation programs is staggering. Nearly 15 billion tablets have been manufactured, packaged, shipped, and distributed. The supply chains established to support such massive operations are enormously complex. With the importance of strong supply chain in global focus like never before, it is an opportune moment to recognize the supply chains that form the backbone of global PC-NTD programs and a unique public–private partnership that was formed to strengthen them.

## The supply chain for donated NTD pharmaceuticals

The NTD pharmaceutical supply chain begins with country-level disease assessments to quantify drug needs for an upcoming time period, followed by submission of drug applications to the approving bodies (drug donation programs and WHO) [4]. Once the requests are approved, they are sent to the manufacturers to begin the production process.

The **“first mile”** supply chain includes sourcing and manufacturing of the active pharmaceutical ingredient (API), processing the API into tablets or pediatric formulations, performing quality control checks, packaging, and shipping to the port of entry of the recipient country or country’s Central Medical Stores (CMS). Upon delivery to the shipping destination, the consignment is inspected, and, once approved, divided into smaller consignments before transport to designated repositories, as determined by the national NTD program.

The “**last mile**” begins when the drugs are transported from regional stores to district-level stores then on to MDA distribution points. Post-MDA, treatment data along with leftover or unusable stock are reconciled according to country policy. Data on all distributed, leftover, and unusable drugs are applied to the country’s quantification process for the following year’s drug request.

Initially, each donation program worked independently in establishing its supply chain processes and dealing with the complexities and challenges of scale up. The lack of collaboration among the donation programs resulted in lost opportunities to improve efficiencies through systematically tackling challenges common across the programs. The development of WHO PC Joint Application Package (JAP) in 2012 and the subsequent consolidation of most donation programs within WHO provided the impetus needed to form the missing connections among industry partners. These connections solidified into a platform for engagement now known as the NTD Supply Chain Forum.

## NTD Supply Chain Forum

The NTD Supply Chain Forum (SCF) is a public–private partnership that was established in 2012 to serve as the platform for engagement for NTD supply chain experts from WHO, pharmaceutical companies, nongovernmental organizations, donor organizations, ministries of health, and logistics providers. Its mission is to support endemic countries in the control/elimination of NTDs by strengthening supply chains to improve access to drugs and diagnostics. While the Forum has historically been focused on PC-NTDs, opportunities to expand into the other NTDs are currently under evaluation.

From an initial 8 organizations, the Forum has expanded to include 17 partner organizations working together to strengthen the supply chains for donations from GSK, J&J, Merck KGaA, Eisai, Pfizer, and MSD. It is a unique example of a successful cross-disease partnership working together with WHO to produce tangible solutions that have significant impact on the delivery of NTD interventions.

## Challenges and solutions

Initially, most of the pharmaceutical donors shipped through DHL Global Forwarding, but the Forum was able to work with DHL to create a dedicated Control Tower within the DHL Global Humanitarian Logistics Competence Centre based in Dubai to serve as a single coordinating body responsible for handling all of the logistics for the drugs donated by GSK, Merck KgaA, and J&J. The Control Tower allows the donation programs to benefit from solutions available only to humanitarian shipments, including 45 days of demurrage-free storage for dry containers and preferential shipping lines. The DHL Control Tower also provides the following:

- a centralized platform to handle orders, contracting, invoicing, process optimizations, reporting, and communications;
- enhanced visibility into shipping processes to reduce the need for intensive tracking on the part of the pharmaceutical donors and WHO; and
- a high degree of knowledge and experience in handling specific products and customized solutions such as optimization of shipping routes.

The development of the DHL Control Tower allowed for significant improvements to the importation process. Initially, most of the pharmaceutical donors shipped drugs to the port of entry, leaving national programs to manage customs clearance and delivery. The resulting loss of visibility and control, intense resource requirements from the countries and their supporting partners, and the high costs of customs delays prompted GSK, J&J, and Merck KGaA to shift from door-to-port to a door-to-door (i.e., the CMS) delivery structure managed through the DHL Control Tower. This shift allowed the pharmaceutical donors to be involved continuously from manufacturing to delivery at the CMS, leading to significant cost savings for the donors as well as overall improved accountability, efficiency, and transparency.

The harmonization of supply chain processes created an opportunity to capture data across programs. NTDeliver (www.ntdeliver.com) was developed to serve as a centralized information system for data related to donated diethylcarbamazine, albendazole, mebendazole, praziquantel, and azithromycin from the initial purchase order (PO) issuance to delivery. It was created to increase visibility and provide real-time data to allow for more flexible supply chain management. The data available in NTDeliver enable the systematic identification of weak points and delays, reduce the need for manual communication, and allow for improved coordination across partners.

The addition of publically available country pages in 2019 proved to be a turning point in the utility of NTDeliver as a tool for national NTD programs. The country pages provide a country-specific summary of all POs in the NTDeliver system. Users are able to obtain critical information such as batch number, expiration date, quantity, estimated and actual arrival dates, estimated and actual delivery dates, and planned MDA dates. Users can also subscribe to country pages to receive weekly updates on changes in shipping status. Access to real-time information on shipments allows national program managers to better plan and prepare for MDAs.

Indeed, data available in NTDeliver show a 1-month reduction in the average total supply chain timeline and a 37% increase in on-time delivery between 2018 and 2019 [5]. While the causes of these improvements are still under investigation, early results from an ongoing research study empirically assessing the impact of NTDeliver on supply chain performance suggest a positive impact on 3 key performance indicators: PO timeliness, arrival timeliness, and delivery timeliness (personal communication, August 2020).

Data collected through NTDeliver have brought to light a number of critical elements in need of improvement. For example, in 2018, NTDeliver showed that 56% of all POs were issued in August and November [5]. The concentration of demand within such a small time frame overwhelmed the capabilities of some manufacturers. Therefore, the SCF worked with WHO and several national program managers to encourage a more even distribution of drug requests. As a result, the number of POs issued in 2019 was much less concentrated, with only 26% issued in the 2 peak months.

NTDeliver is also being used to reduce drug expiration and wastage. Between January 1, 2020 and May 30, 2020, there were 83 batches representing 69,517,000 tablets planned to expire in 8 countries. To improve monitoring and reduce loss due to expiration, an expiry alarm was created in NTDeliver and rolled out at the beginning to 2020 to notify donors, WHO, implementing partners, and country staff when expirations are forthcoming. Those messages are delivered via email 12 months, 6 months, 3 months, 2 months, 1 month, 2 weeks, and finally on the actual expiration date. Currently, expiration reporting can only be used to identify batches “at risk” of expiration due to limitations in last mile inventory reporting. It cannot be used to report on the number of batches or tablets that have expired, although this is a goal for future development.

## Future direction of the NTD SCF

The Forum’s coordination efforts have resulted in significant strengthening of the first mile pharmaceutical supply chain, though more work is needed to ensure countries receive their requested drugs on time. Part of this effort will be to promote stronger inventory management and improved customs clearance processes. Work is already underway to create a repository of country-specific guidance that outlines customs clearance processes, documentation requirements, estimated timelines, and other key details based on data available in NTDeliver and the experiences of SCF members. A standard operating procedures (SOPs) toolkit is being developed to provide detailed guidance on the application process, shipment and storage of drugs, management of waste, forecasting, and other key issues in the first mile and last mile supply chain. Once finalized, all of these resources will be available through NTDeliver.

In an effort to help strengthen in-country supply chains, the Forum is working to identify existing in-country tracking systems that can be integrated with the NTDeliver platform. Such integrations would allow shipping data to be imported directly into country databases to promote country ownership and allow for more comprehensive forecasting, planning, and reporting at the country, regional, and international levels.

During the next phase of work, the Forum will expand its focus to include NTD diagnostics supply chain. Opportunities are being evaluated to adapt and apply the successes and lessons learned from the NTD pharmaceutical supply chain. A landscape analysis will define specific challenges within the current diagnostics supply chain to identify areas where the Forum is uniquely positioned to take action.

Finally, the SCF will continue to serve as a key advocate for strengthening supply chains for NTD products. Though often overlooked, strong supply chains are the backbones of these programs. The 2030 NTD Roadmap has identified supply chain management as 1 of 4 priority action areas for the coming years. The NTD SCF is poised to address this broadening global concern and build on its own momentum to help develop the increased capacity needed to meet this critical programmatic challenge.
